# Silica-Based Bioactive Glasses and Their Applications in Hard Tissue Regeneration: A Review

**DOI:** 10.3390/ph14020075

**Published:** 2021-01-20

**Authors:** Nuha Al-Harbi, Hiba Mohammed, Yas Al-Hadeethi, Ahmed Samir Bakry, Ahmad Umar, Mahmoud Ali Hussein, Mona Aly Abbassy, Karthik Gurunath Vaidya, Ghada Al Berakdar, Elmoiz Merghni Mkawi, Manasa Nune

**Affiliations:** 1 Department of Physics, Faculty of Science, King Abdulaziz University, Jeddah 21589, Saudi Arabia; nallhibi0001@stu.kau.edu.sa (N.A.-H.); emrzog@kau.edu.sa (E.M.M.); 2 Fondazione Novara Sviluppo, 28100 Novara, Italy; Hibamedic@outlook.it; 3 Lithography and Devices Fabrication & Device Research Group, Deanship of Scientific Research, King Abdulaziz University, Jeddah 21589, Saudi Arabia; 4 Operative Dentistry Department, Faculty of Dentistry, King Abdulaziz University, Jeddah 21589, Saudi Arabia; hbakry@kau.edu.sa; 5 Conservative Dentistry, Alexandria University, Alexandria 21526, Egypt; 6 Department of Chemistry, Faculty of Science and Arts, Najran University, P.O. Box 1988, Najran 11001, Saudi Arabia; ahmadumar786@gmail.com; 7 Promising Centre for Sensors and Electronic Devices (PCSED), Najran University, P.O. Box 1988, Najran 11001, Saudi Arabia; 8 Department of Chemistry, Faculty of Science, King Abdulaziz University, Jeddah 21589, Saudi Arabia; mahussein74@yahoo.com; 9 Department of Chemistry, Faculty of Science, Assiut University, Assiut 71516, Egypt; 10 Department of Orthodontics, Faculty of Dentistry, King Abdulaziz University, Jeddah 21589, Saudi Arabia; monaabbassy@gmail.com; 11 Dental Department, Alexandria University, Alexandria 21614, Egypt; 12 Manipal Institute of Regenerative Medicine (MIRM), Manipal Academy of Higher Education (MAHE), Allalasandra GKVK Post, Yelahanka, Bengaluru 560065, India; karthikgvaidya@gmail.com (K.G.V.); manasa.nune@manipal.edu (M.N.); 13 Department of Dentistry, University Medical Services Center, King Abdulaziz University, Jeddah 21589, Saudi Arabia; ghadedoezat@gmail.com

**Keywords:** regenerative medicine, hard tissue regeneration, bioactive glasses, osteoconductivity, osteostimulation, medical applications

## Abstract

Regenerative medicine is a field that aims to influence and improvise the processes of tissue repair and restoration and to assist the body to heal and recover. In the field of hard tissue regeneration, bio-inert materials are being predominantly used, and there is a necessity to use bioactive materials that can help in better tissue–implant interactions and facilitate the healing and regeneration process. One such bioactive material that is being focused upon and studied extensively in the past few decades is bioactive glass (BG). The original bioactive glass (45S5) is composed of silicon dioxide, sodium dioxide, calcium oxide, and phosphorus pentoxide and is mainly referred to by its commercial name Bioglass. BG is mainly used for bone tissue regeneration due to its osteoconductivity and osteostimulation properties. The bioactivity of BG, however, is highly dependent on the compositional ratio of certain glass-forming system content. The manipulation of content ratio and the element compositional flexibility of BG-forming network developed other types of bioactive glasses with controllable chemical durability and chemical affinity with bone and bioactivity. This review article mainly discusses the basic information about silica-based bioactive glasses, including their composition, processing, and properties, as well as their medical applications such as in bone regeneration, as bone grafts, and as dental implant coatings.

## 1. Introduction

Regenerative medicine (RM) is a modern non-operative treatment solution that utilizes the body’s natural healing process to rebuild damaged tissue, heal injuries more effectively, and eliminate pain. This field aims to greatly influence and improve tissue repair and restoration and assist the body to heal and recover [1]. Tissue engineering is a field that utilizes cells, make scaffolds, and supplement with growth factors to aid in tissue regeneration and restore the healthy tissues from damaged or diseased tissues. Tissue engineering and regenerative medicine (TERM) is a multidisciplinary science that is based on establishing three-dimensional (3D), biocompatible, and biodegradable biomaterials that can function as living tissue and may be used to repair or regenerate injured tissue or organs. It involves the knowledge of various fields such as cell biology, materials science, biomechanics, and medical sciences [2].

When grafted within the body, all biomaterials induce tissue responses, whose nature and extent is dictated by the bioactivity and biocompatibility of the biomaterial [3]. Biomaterials are being used for a broad range of applications such as medical implants, artificial joints, dental implants, and devices that stimulate nerves. Examples of biomaterials include metals, ceramics, glass, and polymers. Biomaterials may be distinguished from other materials in that they possess a combination of properties, including chemical, mechanical, physical, and biological properties that render them suitable for safe, effective, and reliable use within a biological environment.

There is an increasing trend for biomaterial to shift from traditional bio-inert material to a new generation of biomaterials, the bioactive materials. A bioactive material can be defined as a material that prompts a specific biological response between the material and the tissue that leads to the development of a bond between them and induce a response within the biological system [4]. Bioactive glasses (BGs) are being used for bone tissue engineering applications and they form a very good material to produce scaffolds for bone regeneration. This is because they have reasonable mechanical strength and hence can withstand stress, they do not undergo corrosion, and they are biocompatible and biodegradable. These properties can be altered on the basis of the application by varying their composition [5,6]. Certain BGs are observed to form a mechanically strong and firm bond with the bone, and some compositions have also been observed to bond well with soft tissues as well as bone. BGs can be used for efficient bone tissue engineering applications as they can enhance revascularization, osteoblast adhesion, enzyme activity, and differentiation of mesenchymal stem cells [5,7,8]. This is possible due to an important characteristic of BGs of time-dependent, kinetic surface modification that occurs post-implantation. The surface of the bioactive glasses forms a biologically active hydroxyl carbonate apatite (HCA) layer, which is chemically and structurally very similar to the bone’s mineral phase and provides an interface that bonds with the tissues [9].

## 2. Silica-Based Bioactive Glasses (BGs)

Bioactive glasses (BGs) were first introduced by Larry L. Hench and his co-workers at the University of Florida in the late 1960s [10,11]. The first artificial inorganic material that had the ability to bond with living bone tissue and form stable and tightly bound interface was the Bioglass. This was composed of a quaternary oxide system consisting of SiO_2_–CaO–Na_2_O–P_2_O_5_ [12]. The application of BGs in humans is not limited to bone implants but there are certain combinations of BGs that can be utilized in both soft tissue restoration and delivering pharmaceutical compounds [5,6,13,14]. BGs have gained a great amount of attention in the field of biomedical science owing to their ability to enhance angiogenesis and osteogenesis [10,15]. BGs are extensively used in the field of hard tissue engineering due to their osteoconductivity, osteo-inductivity, and osteo-integrativity, which are critical for optimal regeneration of bones [16].

The main component in silica-based BGs is silicon dioxide and they are made up of three other basic components: sodium dioxide, calcium oxide, and phosphorus pentoxide [17]. The composition of BGs is crucial in determining their properties and characteristics. Depending on the composition, some BGs are able to form bonds with soft tissues and bone, some can bind only to bone, some are not able to form a bond at all, and others get resorbed completely within a few weeks [5,6]. Over the years, there has been development of several types of bioactive glasses. These can be broadly classified as silicate glass (Bio glass or 45S5 bioactive glass), glass ceramics (Bon Alive or S53P4 bioactive glass), and silica-based glasses such as 13-93 and 13-93B1, among others [17,18,19,20,21,22,23,24,25,26]. The composition of these three types of bioactive glasses is shown in Table 1 below. Bioglasses can also be divided into the following two categories [22]: (i) Class A bioglasses, which are osteoproductive, and they bind with both soft tissues and bone; the HCA layer is formed within several hours, and (ii) Class B bioglasses, which are osteoconductive, and their bonding with soft tissues is not facilitated; the HCA layer takes a few days to be formed.

### BG Properties

**Figure 1:**
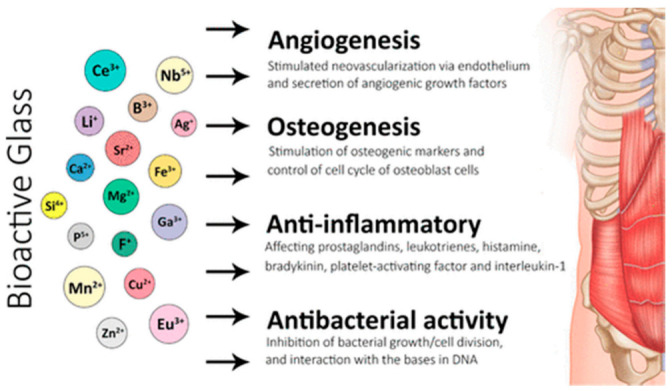
Applications of bioactive glasses in angiogenesis, osteogenesis, anti-inflammatory and anti-bacterial activities [28].

On implantation, Bioglass interacts with its biological surrounding and elicits a specific biological response, for example, the hydroxyapatite layer formation between the tissue and material [27]. This displays the bioactivity of BGs. Figure 1 depicts the applications of bioactive glasses in angiogenesis, osteogenesis, anti-inflammatory and anti-bacterial activities [28].

The most bioactive glass has a superior surface area with a higher dissolution rate and thus a faster apatite formation [29]. Hench, in 1980, reproduced the in-vivo formation of the Hydroxyapatite (HAP) layer in Tris buffer solution at a pH of 7.4. It was also confirmed independently by Kokubo and Hench that apatite could be formed on the Bioglass surface in simulated body fluid (SBF) [30,31].

**Figure 2:**
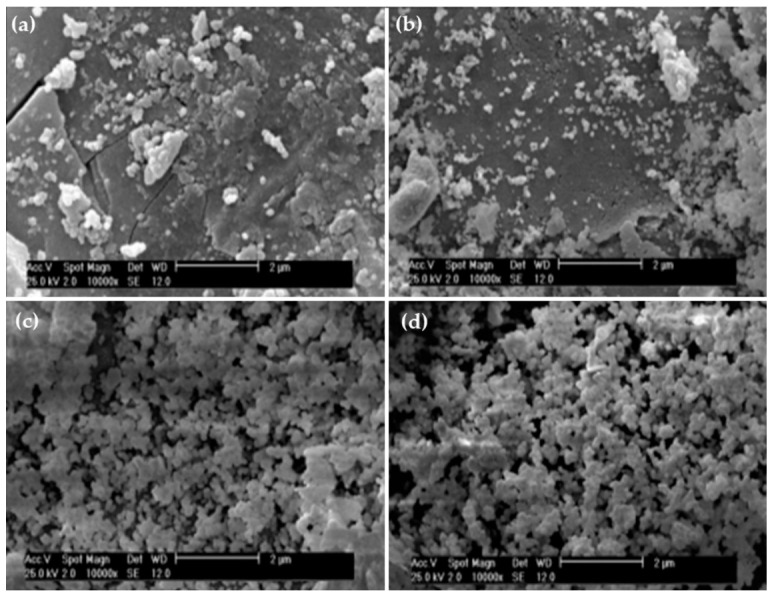
SEM micrographs of S6 BG sample after soaking in the simulated body fluid (SBF) solution for (**a**) 3 days, (**b**) 7 days, (**c**) 14 days, and (**d**) 21 days [32].

Goudarziet al. (2020) [32] investigated the bioactivity and proliferation of G292 cells using strontium-doped BGs. Figure 2 shows the process of hydroxyapatite formation at different time points of immersion of the S6 (SrO 6 mol%) sample. With increase in time, the density of HA particles was also observed to increase (Figure 2). It is seen that on the 21st day of soaking, the surface of BG was completely covered with hydroxyapatite [32].

The ability of BGs to demonstrate antibacterial activity is considered to be one of the most attractive characteristics needed in regenerative medicine. This antibacterial effect can be due to the following three principles: (i) the dissolution of surface (alkali) ions that cause an increase of pH and a higher osmotic pressure [33]; (ii) the ability of doping antibacterial elements, such as silver, copper, or zinc to BGs [34,35,36,37,38,39,40,41]; or (iii) loading antibiotic into BGs, which is eluted out during degradation [17].

Table 2 summarizes the main mechanical properties of some of the commercially available BGs and glass ceramics, human bones, and hydroxyapatite [42]. Doping BGs with certain metallic ions such as Ag, Sr, Fe, Mg, Zn, and Mn have been observed to enhance their mechanical properties [43]. Mechanical properties of BGs can be increased by addition of fluorine and nitrogen. Fluorine induces considerable reductions in glass melting temperatures (Tm) and glass transition temperatures (Tg), while incorporation of nitrogen increases elastic modulus and hardness [44].

Sr-doped BGs promote osteoblast proliferation and also decrease the osteoclast activity in the cell culture [45]. The rate of release of ions from a biomaterial at the defect site is decreased in the presence of Sr, as indicated from a study by Kargozar et al., which has therapeutic benefits [46]. Erol-Taygun et al. demonstrated that patients suffering from osteoporosis can be greatly benefited with the use of Sr-doped BGs [47]. Zinc ion can improve bone-bonding of glass; inhibit bone resorption; control cell growth, differentiation, and development; and stimulate synthesis of protein [48]. Lithium plays a constructive role in angiogenesis and osteogenesis. Khoramiet al. reported the controlled and localized release of Li ions from BGs represents a promising alternative therapy for bone regeneration [49]. Specific properties of BGs can also be enhanced and controlled when synthesized at a nanoscale, including their biocompatibility and bioactivity. Interestingly, BGs in nanoscale demonstrate a better osteo conductivity as compared to normal bulky BGs [50].

## 3. Medical Applications of BGs

Bioglass is most commonly used for bone grafts. BGs help in the repair of hard tissues and are being synthesized in various compositions for preparation of scaffolds, coating materials for implants, and other applications [51,52]. BGs have also been used for dental air polishing, yielding better results in terms of stain removal and patient comfort when compared to traditional sodium bicarbonate powder. BG can also be utilized for cutting cavities in teeth by air abrasion [53]. A few decades ago, these glasses modified the functions and capabilities of biomaterials from bio-inert to bioactive by stimulating a strong response after implanting in the human body [54]. Some of the common uses of BGs are discussed here.

### 3.1. Bone Regeneration

Bone regeneration is a complex process that can be seen during the healing of a normal bone fracture. Continuous remodeling of the bone takes place throughout adult life. However, there are complex clinical conditions in which bone regeneration must be achieved in great amounts, such as for skeletal reconstruction of large bone defects created by trauma, tumor resection, infection, and skeletal abnormalities [55]. Different bone substitutes are being used that can be derived either from biological products such as platelet-rich plasma, demineralized bone matrix, hydroxyapatite, etc., or synthetics such as bioactive glasses, calcium sulphate, tri-calcium phosphate ceramics, or polymer-based substitutes. These substitutes must be chosen on the basis of their intended use [56].

As BGs are non-crystalline ceramics that can bond to living tissues and also stimulate the growth of new tissue while degrading over time, they are considered valuable candidates for tissue engineering applications [57]. Initially designed to fill bone defects, BGs such as Bioglass, which can bind to both soft and hard tissues, are now being used for a wide range of clinical purposes. However, due to their high tendency to crystallize during thermal treatments, poor mechanical strength, and high brittleness, their widespread applications have been limited [58]. To overcome these limitations, researchers have mixed a variety of polymers with powders or granules of BGs with the aim of producing hybrid composites with the desired biological and mechanical properties specific for a clinical application [57]. Natural polymers, such as collagen, have been used. Due to its superior biocompatibility, biodegradability, wound-healing properties, and low immunogenicity, collagen is the protein of choice. There have been several reports of collagen-based BG composites, with a focus on bone regeneration [57,59].

**Figure 3:**
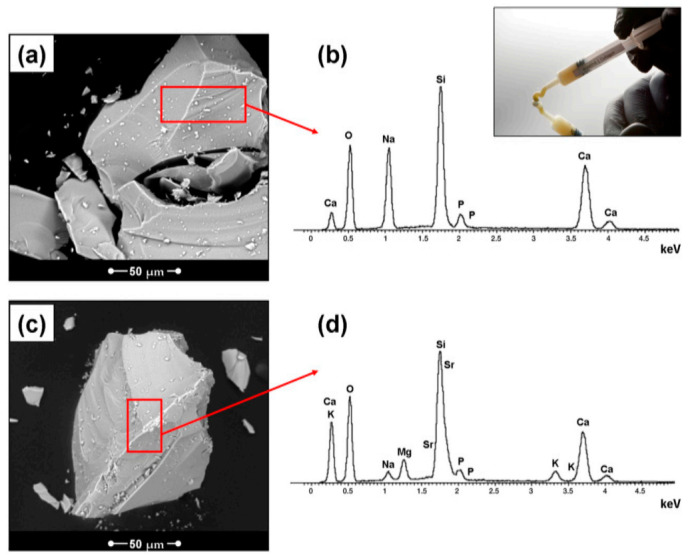
Morphological evaluation of the Bioglass (**a**) and BGMS10 (**c**) granules; (**b**,**d**) EDS results; inset: a BGMS/C putty syringe [57]. The red box, explain the selected area from the smaple for the EDX analyses

In a study by Belluci et al. [57], granules of an innovative bioglass, BGMS10 (Composed of (mol%): 2.3 Na_2_O, 2.3 K_2_O, 25.6 CaO, 10.0 MgO, 10.0 SrO, 2.6 P_2_O_5_, 47.2 SiO_2_), were blended with collagen. Polyethylene glycol (PEG) was added as a binder to obtain a BGMS/C composite putty suitable for oral and dental applications. The applications also include mucosal injury, bone defects, and periodontal pockets [57,60]. BGMS10, containing magnesium and strontium, was observed to be very promising due to its ultrahigh crystallization temperature and enhanced bioactivity [57]. A comparative study of 45S5/collagen putty (45S5/C) and BGMS/C, prepared with the same proportions of glass, collagen, and PEG, was also performed [57]. Figure 3 reports the morphological evaluation of the Bioglass and BGMS10 granules, along with their composition (by EDS analysis), when tested using murine fibroblasts. It was observed that the BGMS10/C showed more promising results with enhanced cell proliferation [57]. It should be stressed that although in recent years many collagen/bioglass composites have been developed, most of them are porous scaffolds for bone tissue engineering. On the contrary, there is a lack of moldable putties or injectable composites for dental applications.

### 3.2. Bone Grafts

Bone grafting is a surgical procedure that replaces a missing bone with a material that is derived either from patient′s own body, or an artificial or natural substitute [55]. Bone tissue has the ability to regenerate completely, given the space into which it has to grow, and this makes grafting of bone possible [61]. Bone grafts are employed as scaffolds and fillers to promote wound healing and facilitate bone reformation. The graft material is generally replaced by the natural bone as it grows, which results in a fully integrated region of the new bone [57]. A bone graft material is required to be osteoconductive. Bioactive materials with controlled release of biochemical stimuli help in repairing diseased or damaged tissue with a more biologically based approach [62]. BG bone grafts are based upon the concept of in situ regeneration of bone with structure, architecture, and mechanical strength equivalent to normal cortical and cancellous bone.

BGs have been used as a bone graft for several years now [63]. BGs have superior osteoconductive properties and they also stimulate the growth of new bone over their surface [64]. Oonshi et al. [65] conducted a study to compare the properties of hydroxyapatite (HA) and BG when they are used as a bone graft in an animal model. They concluded that BGs are not only easy to manipulate, but that they were also able to restore the bone within 2 weeks, whereas HA took 12 weeks to produce an equivalent response. It was also concluded that the use of BG as a bone graft demonstrates excellent bone healing properties [65,66,67]. Bioglass has been clinically used as a synthetic bone graft material for over 10 years. It has been used under two different product names: Nova bone for orthopedics and Perio-glass for maxillofacial surgery [65,68,69]. In 2005, the Food and Drug Administration (FDA) approved the osteostimulatory effect of Bioglass [70]. Traditional osteoconductive bioceramics do not have osteostimulatory effects, while Bioglass has both osteoconductive and osteostimulatory effects [68,71,72]. Thus, it is highly favorable for the structure and the composition of bone substitutes to allow vascularization, with an interconnected porosity and a favorable biochemical support. This accelerates the bone remodeling by facilitating colonization and retention of osteogenic cells and nutrients through an enhanced capillarity [73]. The establishment of a vascular network will aid in supplying nutrients, soluble factors, and minerals such calcium and phosphate, which are crucial for the bone healing process [74,75].

### 3.3. BG Implant Coatings

Dental implants (DI) are screw-shaped devices that are inserted in the alveolar bone in order to support prosthodontic constructions to improve function and appearance [76]. Direct contact of the implant surface with the bone tissue is required for achieving adequate retention in bone and osseointegration. BGs can aid in bonding the implant with the bone, providing antimicrobial protection and thus reducing the total time required for the treatment [77]. As BGs can produce favorable biological response, after they are in contact with surrounding fibro-osseous tissues, they are seen as a favorable material [78].

One of the most widely adopted and conventional methods of improving surface bioactivity and biocompatibility is the process of surface coating. BGs are highly biocompatible and have a better chance of bonding and integrating with human tissue than the metal implants, making them a good option for improving the bioactivity and biocompatibility of these metals. If the implant surface fails to integrate firmly with the host tissues, fibrous tissue would develop at the interface, and this would lead to loosening of the implant, which ultimately results in the failure of the implant [79]. As the metallic implants are bio-inert, they get encapsulated with fibrous tissue after implantation and fail to attach with the tissue, which poses a serious need for implants to have bioactive coatings [80]. BGs offer a wide range of benefits such as (i) replacing damaged tissue and bone that will integrate well with the body’s environment, (ii) facilitating regeneration of tissue, and (iii) degrading at a rate similar to the rate of regeneration of tissue. In terms of coating BGs on metal implants, they can form hydroxyapatite at the interface of the implant and host tissue, which facilitates in better integration of the implant [79]. Furthermore, they can also regulate or inhibit corrosions of the metallic implant in the biological system. The glass matrix can maintain its glass character and its chemical and physical properties even after doping them. Many new glass compositions have been proposed and found several biomedical applications including dental fillings and coatings [79]. This composition flexibility allows us to introduce additional functionalities such as enhancement of osteo growth by doping with Sr^2+^ and angiogenesis by doping with Cu^2+^ [79,81]. BGs can be easily coated on metallic dental implants, such as on titanium implants with screw threads. However, it must be made sure that the thermal expansion coefficients of the glass and the metal match in order to prevent the glass from being pulled away from the metal while processing [82,83]. For instance, the thermal expansion coefficient of the Bioglass and titanium do not match. In order to tackle such a problem, the Na_2_O and CaO of the Bioglass (SiO_2_–CaO–MgO–Na_2_O–K_2_O–P_2_O_5_) system are replaced with K_2_O and MgO, respectively, in order to modify the thermal expansion coefficient [84]. Another example is coating with the following composition (by weight): 53% SiO_2_, 6% Na_2_O, 22% CaO, 11% K_2_O, 5% MgO, 2% P_2_O_5_, and 1% B_2_O_3_ on titanium implants [85].

Studies have shown that compared to non-coated implants, coated implants saw more bone growth on them. Using appropriate compositions, the mismatch of thermal expansion coefficients can be made to match, and BGs can be successfully used as coatings. The use of BG as a coating material for dental implants has produced better results in terms of adherence to the metal surface of implant and bone regeneration; however, more research is still needed in this area.

### 3.4. Enamel Re-Mineralization

Re-mineralization of the dental hard tissues (enamel and dentin) is a challenging problem faced by dentists across the globe on a daily basis [86,87]. The available technologies require to be applied repeatedly and take a long time to achieve significant re-mineralization of the dental hard tissues [86,87]. Orthodontic patients have increased incidence of demineralization because of the increased bacterial counts in their saliva [88]. Many research groups have focused their efforts to design materials and agents capable of efficiently re-mineralizing enamel and dentin within short duration [86,87]. The challenge faced by researchers in the field of dentistry to employ BGs as re-mineralizing agents was the absence of enamel-forming ameloblast cells, which are normally lost during the eruption of the human teeth and are lacking in the mature enamel [89]. Studies have suggested that adding an acidic medium to the bioactive glasses can boost their bioactivity and result in releasing large amounts of calcium and phosphate ions onto the dental hard tissues, which results in their re-mineralization [86,87,90,91,92,93,94,95,96].

**Figure 4:**
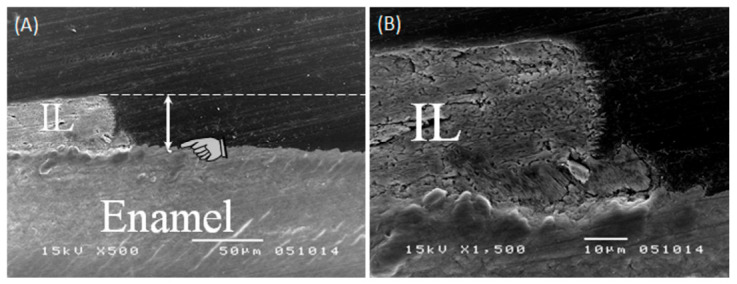
45S5 bioactive glass re-mineralization for the enamel process. (**A**) Finger pointer pointing to the original enamel surface. Dashed line showing the level that the interaction layer (IL) formed by Bioglass (can reach approximately 40 microns). (**B**) The interaction layer is integrated with the surface of enamel with no gaps observed at this magnification (courtesy of Dr. Ahmed Samir Bakryand Dr. Mona Aly Abbassy).

Recently, BGs such as Bioglass [93,94,95,96] and FBG (fluoride bioactive glass) [90] have shown promising results in re-mineralizing dental enamel and dentin. Studies have shown that these materials can form a bioactive film mainly consisting of phosphate and calcium on top of the enamel. This bioactive film was found to be resistant to abrasion and erosion and hence can be used as orthodontic sealers (Figure 4). It has been suggested that bioglasses can possibly be applied as dentin desensitizers, re-mineralizing agents, or as a bioactive temporary filling material [86,87,88,89,90,91,92,93,94,95,96,97].

Dentin hypersensitivity (DH) is one of the most common clinical conditions usually associated with exposed dentinal surfaces. Dentin hypersensitivity is caused by the exposure of the dentinal tubule, and with the increasing diameter of the tubule, dentin is demineralized by micro-organisms within the oral cavity or by acidic food. Thus, the key point of DH treatment is to desensitize dentin through the re-mineralization of the exposed dentin by obstructing the exposed dentinal tubules [98]. Bio-silicate (P_2_O_5_–Na_2_O–CaO–SiO_2_), A fully crystallized bioactive glass-ceramic has been proposed to treat DH by depositing hydroxyl carbonate apatite in open dentinal tubules [99].

### 3.5. Treatment of Periodontitis

Periodontium is a biologically complex structure that supports the human teeth. It consists of gingiva, periodontal ligaments, cementum, and alveolar bone. Periodontitis is one of the most common acute or chronic inflammation of periodontal structures encountered in dental clinics. It is mainly initiated due to the bacterial biofilm presence at the dental structure causing a destruction of the periodontium and resulting in gingivitis and pockets formation between the tooth roots and the overlying gingival. Theses pockets are characterized by loss of periodontal ligaments (PDL) and alveolar bone resorption with or without gingival recession [100]. Eventually, periodontitis results in tooth loosening and, consequently, tooth loss [101]. The improvement of periodontal disease prognosis requires the application of biomaterials that both stimulate the periodontal tissue repair and prevent bacterial accumulation. BGs, which are well known for their osteogenic stimulatory effect, have also been shown to have antimicrobial impacts when implanted in areas of periodontal defects, or applied as topical endodontic disinfectants with no effects on dentin stability [102,103,104]. Thus, a special concern has been given to BG-containing products such as PerioGlas, which can be packed inside the periodontal defect to stimulate the periodontal bone regeneration, particularly the defective interproximal bone structure [105]. Furthermore, PerioGlashas been involved in periodontal surgery, not only for bone stimulation but also as a hemostatic agent for the trabecular bone hemorrhage [105,106,107]. In endodontic surgery, PerioGlas demonstrates a highly successful regeneration of the apical bone structure [108].

Other BG-containing products such as ERMI (endosseous ridge maintenance implant) have also been considered in periodontal surgery as a means of maintaining the alveolar ridge height from resorption. ERMI is a cone-shaped BG product designed to fit the dental socket immediately after tooth extraction to offer a steady ridge for teeth and to patch up the tooth root. The five-year follow up investigation proved the safety of ERMI clinical application and demonstrated 85.7% cone retention within the socket, which provided a significant support for periodontal structures and the dental prosthetic treatment [109].

**Figure 5:**
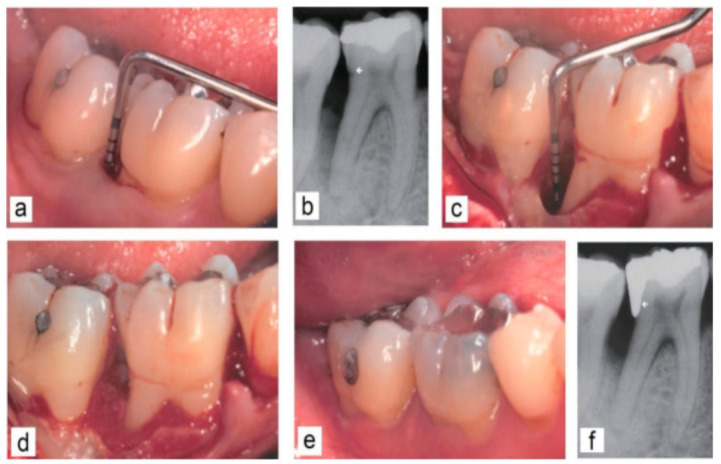
Clinical application of BG in a patient with generalized aggressive periodontitis. (**a**,**b**) Intra-bony defect prior to surgery. The arrow sign on the radiograph indicates distal cervical caries and the location of the cemento-enamel junction as a landmark. (**c**,**d**) Intraoperative situation after exposure of the defect. (**e**,**f**) Clinical and radiological situation 12 months after surgery. Moreover, the distal cervical caries was treated with a new filling [78].

Another clinical study demonstrated the effectiveness of BG application in patients with generalized aggressive periodontitis. Post-surgery, 6- and 12-month clinical results demonstrated a reduced depth of the periodontal pocket by probing and improved periodontal attachment level. This suggests the potential of BG not only for stimulation of bone repair but also for the long junctional epithelium formation [110]. Figure 5 shows the clinical application of BG in a patient with generalized aggressive periodontitis [78].

Moreover, BG demonstrates significant improvement of the signs related to gingivitis. In Vivo studies have demonstrated that significant reduction in gingival bleeding and supra-gingival plaque formation can be achieved by a dentifrice containing Nova Min as compared to a placebo dentifrice [111]. In another study conducted on human subjects with gingivitis, the topical application of BG reduced the signs of gingival inflammation [112,113].

### 3.6. Wound Healing in Dentistry

**Figure 6:**
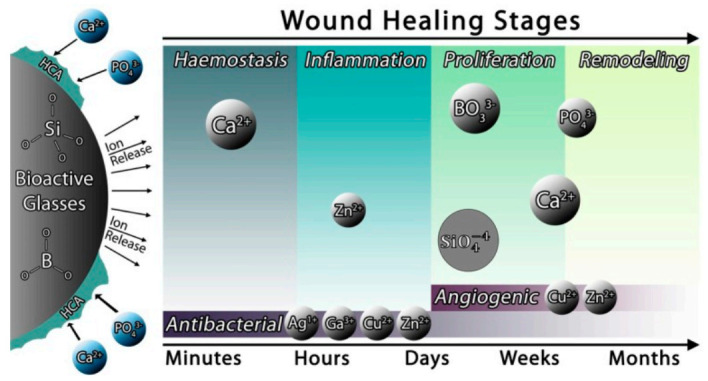
The release of some metal ions from BGs into the surrounding environment has a positive effect on wound healing [116].

The wound healing process is highly complex, mainly involving three overlapping phases, namely, inflammation, proliferation, and remodeling. Any disruption leads to abnormal wound healing [114]. Procedures that dentists perform such as exodontia rely on adequate wound healing. Some of the BGs, such as the silicate-based BG (Bioglass)and borate-based 13-93B3, have been shown to have enhanced the wound healing process due to their ability to release ions that can stimulate various processes such as hemostasis, antibacterial efficacy, and angiogenesis, amongst others. A wound dressing made of a borate-based glass has recently received regulatory approval for use in the treatment of acute and chronic wounds [115]. BGs enhance wound healing mainly by releasing various therapeutic ions from their structure (Figure 6) [116]. The capability of BG to stimulate soft tissue regeneration relies on its enhancement of collagen deposition and angiogenesis during the process of wound healing [117]. In vitro and in vivo experiments have demonstrated that the ionic release of BG causes macrophage activation to the M2 phenotype in addition to stimulating macrophages for more anti-inflammatory and angiogenic growth factor expression [117]. The in vitro cultures of macrophages with BG revealed an accelerated fibroblast and endothelial cell migration in addition to the fibroblast stimulation to express more proteins and growth factors such as fibronectin, collagen type I, basic fibroblast growth factor (bFGF), epidermal growth factor (EGF), and vascular endothelial growth factor (VEGF) [117] with the subsequent increase of extracellular matrix protein deposition and the formation of capillary-like endothelial networks (D). Furthermore, the in vivo experiments demonstrated that BG application at the wound site demonstrated a reduction of the inflammation, manifested as more M2 macrophage phenotype and less neutrophils during initial healing stages in addition to the enhanced blood vessel formation and fibroblasts differentiation into myo-fibroblasts [118] with the subsequent acceleration of wound closure [117].

Clinical studies that involved generalized aggressive periodontitis cases demonstrated that the application of BG in the deep intra-bony pockets resulted in a statistically significantly reduced pocket depth 6–12 months after surgical intervention, with the five-years results being shown to be radiographically optimal [119].

In biphasic dental implant procedures, dental implants are generally placed either at the same level as the surface of the bone or directly under it. It is between the prepared osteotomy edge and the edge surface of the implant that the healing of the bone occurs [120]. Depending on the mechanical stress caused by occlusal forces, bone remodeling around the dental implant persists for at least 1year. BGs inducing active bio mineralization in vivo have been in high demand in the development of clinical regenerative medicine. The replacement of tissues demands very high importance in this technological era. The scope of application of BGs in this area is enormous, due to its versatility, and it is something that must be worked upon.

### 3.7. Protection of Dental Pulp

Dental pulp is the essential part of the tooth, consisting of blood vessels and nerves and being responsible for nutrition, sensation, and vitality of the tooth. Pulp chamber is directly surrounded by dentin, and coronal dentin forms the roof of this chamber. Dental pulp might be exposed due to a variety of reasons such as caries, trauma, or mechanical cavity preparation of the tooth during dental treatment. In order to maintain the exposed pulp tissue from bacterial invasion, many biomaterials that induce the formation of reparative dentin over the exposed portion are involved. The reparative dentin forms a calcified tissue, known as dentin bridge, facilitating pulp healing [121]. A variety of biomaterials have been applied for pulp capping such as calcium hydroxide, calcium eugenol, tricalcium phosphate, isobutyl cyanoacrylate, and calcitonin. These materials, however, represent a number of drawbacks and limitations including pulp tissue dystrophic calcification and persistent pulp inflammation, which leads to pulp degeneration [122,123]. In addition to difficult clinical manipulation and low compressive strength causing bulk fractures of the restoration [124], micro-leakage and pulp infection may occur [125]. Thus, a special concern has been directed towards BG as a bioactive material capable of forming a hydroxyapatite surface layer when contacting an aqueous environment. The *in vivo* application of Bioglass over the exposed dental pulp results in the formation of reparative dentin complete bridges covering the entire pulp exposure. In this study, the reparative dentin bridge beneath Bioglass was free of soft-tissue embodiment in comparison to reparative dentin formed under the autologous demineralized dentin matrix (DDM) [126] and hydroxyapatite pulp caps [127]. Moreover, the reparative dentin bridges formed beneath Bioglass pulp caps were tubular, in comparison to those formed under calcium hydroxide (Life), which differed according to the underlying pulp condition. Accordingly, tubular reparative dentin was observed in association with superficial pulp-tissue necrosis, while tubular reparative dentin formed in the absence of pulp-tissue necrosis [126]. Thus, the variety of mechanisms of dental pulp repair in relation to the particular pulp-capping material must be considered in the future clinical application.

### 3.8. Bioactive Glass Application in Surgical Sutures

A variety of surgical sutures are utilized for the purpose of tissue approximation during the procedure of wound closure [128]. A major disadvantage of surgical sutures is the susceptibility to bacterial biofilm adherence at the suture surface or among the braided multi-filaments causing a surgical site infection and healing impairment [129,130,131]. Therefore, much effort has been dedicated to improve the suture antibacterial impact, and hence the antibiotic coatings of sutures have been developed as infection-combating approaches [131]. The prolonged utilization of antibiotics, however, supports the development of bacterial resistance and the surgery site infection as a consequence [132]. Thus, the attempts of finding an antibiotic alternative for coating the surgical sutures have directed the research concerns towards the possibility of coating surgical sutures with bioactive glass as a well-known antibacterial biomaterial [133]. Interestingly, the antibacterial activity of BG is not only determined by the bacterial strain but also by the ionic release and the prompt alteration in the pH of the surrounding environment, which are highly dependent on the BG composition, concentration, and the particle size [134,135]. An interesting in vitro study investigated the BG-coated sutures with different ionic compositions for their efficacy against *Staphylococcus aurous*, *Streptococcus mutans*, and *Lactobacillus* bacteria as predominant oral cavity strains highly related to oral infections [133]. The investigation involved BG-coated sutures of two different types: the original BG (Bioglass) and the multi-component BG that was achieved by adding Zn, Mg, and K into the Bioglass composition. Both BG coatings were of <45 μm particle size.

The results demonstrated that the antibacterial activity of multi-component BG was significantly higher than that of the Bioglass. This noticeable difference in the antibacterial activity could be attributed to the higher content of alkali metals and alkaline earth ions in the composition of multi-component BG, which consequently increases the alkalinity of the surrounding culture medium and thereby demonstrates a higher antibacterial impact [133].

## 4. 3D Printing of Bioactive Glasses

3D bio-printing or additive manufacturing is an evolving technology in which scaffolding materials and cell-laden hydrogels are deposited in a pre-determined fashion to generate 3D porous constructs. Currently, researchers are coalescing materials such as bioactive glasses and advance techniques such as3D printing techniques to generate custom scaffolds with precise pore architectures. Bergmann et al. prepared a composite of β-tricalcium phosphate (β-TCP) and a bioactive glass by the 3Dprinting process, proving that there is promise in the capacity to print custom-made bone substitute implants [136]. Murphy et al. 3D printed human adipose stem cells (ASCs) with a polycaprolactone (PCL)/bioactive borate glass composite, which showed a high potential for bone regeneration [137]. Pires et al. used the 3D printing technique to bio-fabricate scaffolds from T14P43 glass powders that had good mechanical properties suitable for bone tissue regeneration with low load-bearing properties [138]. Qi et al. prepared 3D BG scaffolds by the 3D printing technique and coated their surface with mesoporous bioactive glass (MBG), which retained good osteoinductive and osteogenic properties, making them attractive candidates for bone defect repair [139]. Wu et al. prepared a combination of mesoporous bioactive glass, sodium alginate, and gelatin by a three-dimensional printing technique along with naringin and calcitonin gene-related peptide as drugs to prepare drug-loaded scaffolds. There sustained release hydrogel 3D scaffolds deliver a prospective application for bone tissue engineering [140]. Zhang et al. fabricated the 3Dprinted scaffolds of ternary composites containing mesoporous bioglass fibers of magnesium calcium silicate (mMCS), gliadin (GA), and polycaprolactone (PCL), which displayed tremendous in vivo osteogenesis demonstrating abundant future for bone regeneration [141].

Montalbano et al. tried a combination of type I collagen and strontium-containing mesoporous bioactive glasses to obtain hybrid material with high osteogenic behavior [142]. Baino et al. prepared bioactive silicate glass scaffolds of SiO_2_–P_2_O_5_–CaO–MgO–Na_2_O–K_2_O glass by a robocasting process that supports the possible suitability of the material for bone repair applications [143].

**Figure 7:**
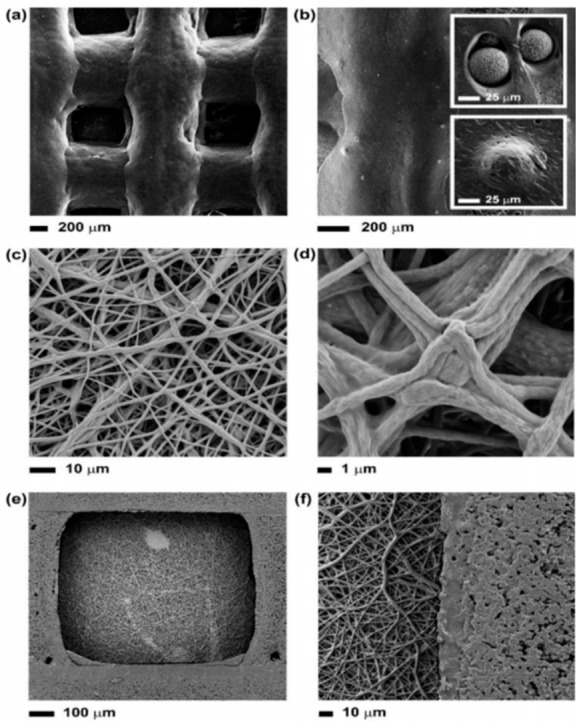
SEM images at different magnifications of (**a**,**b**) the 3D-printed scaffolds, showing also the BG microspheres exposed onto the surface or embedded into the polymer matrix (insets); (**c**,**d**) the surface of the composite scaffold covered with a layer of electrospun polycaprolactone (PCL)– poly (glycerol sebacate) (PGS) mats, pointing out the fusion between fibers; (**e**,**f**) the surface of the composite scaffold without the layer of electrospun fibers [145].

Kolan et al. fabricated scaffolds with five different architectures at different porosities with bioactive borate glass using the selective laser sintering (SLS) process, which demonstrated potential for bone repair [144]. Toure et al. designed multi-layer scaffolds of poly(caprolactone), poly(glycerol sebacate), and bioactive glasses through combination of 3D printing and electrospinning techniques, which has possible applications in tendon and ligament tissue engineering [145] (Figure 7).

## 5. Conclusions

The use of bioactive glasses is a promising field of study that has achieved positive results in various applications. In the last few decades, BGs have been extensively studied and many challenges have been overcome. BG composites have been synthesized to enhance the various necessary properties for BGs to become a better bioactive material. BGs have the potential to revolutionize the field of regenerative medicine, hard tissue regeneration in particular, in the near future. This review article discussed the bioactive glasses in general, including their composition, processing, and properties, as well as their medical applications in hard tissue regeneration.

Furthermore, regenerative medicine in the field of dentistry has had a large number of great achievements in recent years. With more studies and better understanding of BGs, we can possibly achieve complete regeneration of tooth along with its associated tissues, regeneration of bone, and also improved soft tissue regeneration. However, most of these achievements rely on complicated techniques and require long-term studies and *in vivo* testing to confirm its reliability, efficiency, and exact mechanisms of action. With the increase in in vivo studies and positive results, a greater number of healthcare providers will adopt these techniques and technologies in their daily practice.

## Figures and Tables

**Table 1:** Composition of different types of silica-based bioactive glasses [17,19,20,21].

Type	Example of Bioactive Glasses		Composition					
		SiO_2_ (wt%)	Na_2_O (wt%)	CaO (wt%)	P_2_O_5_ (wt%)	K_2_O (wt%)	MgO (wt%)	B_2_O_3_ (wt%)
Silicate glass	Bioglass or 45S5 bioactive glass	45	24.5	24.5	6	–	–	–
Glass ceramic	BonAlive or S53P4 bioactive glass	53	23	20	4	–	–	–
Silica-based glass	13-93 bioactive glass	53	6	20	4	12	5	
	13-93B1 bioactive glass	34.4	5.8	19.5	3.8	11.7	4.9	19.9

**Table 2:** Mechanical properties of bioglass, ceramics, and human bones [42].

Material	Comprehensive Modulus (GPa)	Comprehensive Strength (MPa)	Fracture Toughness (MPa m^1/2^)	Bending Strength (MPa)	Vickers Hardness (MPa)	Structure
HA	35–120	100–150	0.8–1.2	60–120	90–140	Ceramic
Bioglass 45S5	60	-	0.6	40	-	Glass
Bioglass 52S4.6	60	-	-	40	-	Glass
Trabecular bone	0.05–0.6	1.5–7.5	0.1–0.8	10–20	40–60	-
Cortical bone	7–30	100–135	2–12	50–150	60–75	-
